# Theory-Guided Materials Design of Multi-Phase Ti-Nb Alloys with Bone-Matching Elastic Properties

**DOI:** 10.3390/ma5101853

**Published:** 2012-10-15

**Authors:** Martin Friák, William Art Counts, Duancheng Ma, Benedikt Sander, David Holec, Dierk Raabe, Jörg Neugebauer

**Affiliations:** 1 Max-Planck-Institut für Eisenforschung GmbH, Max-Planck-Strasse 1, 40237 Düsseldorf, Germany; E-Mails: wacounts@gmail.com (W.A.C.); d.ma@mpie.de (D.M.); bene-sander@gmx.de (B.S.); raabe@mpie.de (D.R.); neugebauer@mpie.de (J.N.); 2 Department of Physical Metallurgy and Materials Testing, Montanuniversität Leoben, Franz-Josef-Strasse 18, A-8700 Leoben, Austria; E-Mail: david.holec@unileoben.ac.at

**Keywords:** bio-materials, ab initio, Ti alloys, multi-phase composites, multi-scale, finite element method, biocompatibility

## Abstract

We present a scale-bridging approach for modeling the integral elastic response of polycrystalline composite that is based on a multi-disciplinary combination of (i) parameter-free first-principles calculations of thermodynamic phase stability and single-crystal elastic stiffness; and (ii) homogenization schemes developed for polycrystalline aggregates and composites. The modeling is used as a theory-guided bottom-up materials design strategy and applied to Ti-Nb alloys as promising candidates for biomedical implant applications. The theoretical results (i) show an excellent agreement with experimental data and (ii) reveal a decisive influence of the multi-phase character of the polycrystalline composites on their integral elastic properties. The study shows that the results based on the density functional theory calculations at the atomistic level can be directly used for predictions at the macroscopic scale, effectively scale-jumping several orders of magnitude without using any empirical parameters.

## 1. Introduction

The alloys intended for biomedical applications such as for human implants should have a good corrosion stability in the human body, high fatigue resistance, high strength-to-weight ratio, good ductility, low elastic modulus, excellent wear resistance, low cytotoxicity, and a negligible tendency to provoke allergic reactions [1,2,3,4,5,6,7,8,9,10,11,12,13,14]. In the case of bone implants, finding materials that meet all the aforementioned criteria for biomedical applications and have a modulus near that of bone has proven to be a major challenge [15,16,17,18,19,20,21,22,23,24,25,26,27,28]. It is particularly important that the elastic mismatch between the bone replacement material and existing bone be minimized. When the elastically soft bone tissue is replaced by a stiffer implant, the implant takes over a considerable amount of the load, shielding the surrounding parts of the skeleton. Reducing the physiological loads on the bone entails re-sorption processes that lead to a drop in bone density, mineralization state, strength, and health. Stress shielding can finally lead to contact loosening, implant failure, or debris-induced infections [29,30,31,32]. Currently, alloys with hexagonal close-packed (hcp) structure based on *α*-Ti, with a Young’s modulus as high as 120 GPa, are frequently used as implant materials, even though the Young’s modulus of bone is 20–30 GPa. This large elastic mismatch between *α*-Ti and bone has fueled interest in (bcc) *β*-Ti, which have a reduced Young’s modulus of 65 GPa [18,19,20,21,22,23,24,25,26,27]. Therefore, the next generation of bone-replacing materials will likely be based on softer *β*-Ti alloys. In order to identify alloy compositions meeting all above mentioned criteria, an intensive material design effort will be necessary. To reduce both time and cost accompanying such extensive experimental casting and testing, new approaches combining experimental and theoretical methods are sought.

### Theory-Guided Materials Design

**Figure 1:**
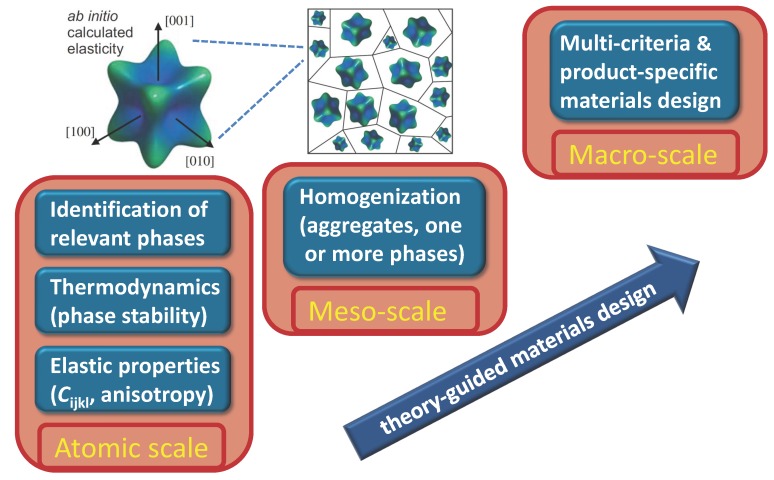
Schematic overview of the multi-scale materials-design strategy combining analysis of the thermodynamic phase-stability; and calculation of single-crystalline elasticity data obtained at atomic level by first-principles calculations with self-consistent homogenization techniques in order to bridge scale differences.

A modern alternative to classical metallurgical concepts is a theory-guided materials design (TGMD). The concept combines (i) quantum-mechanical calculations of thermodynamic stability and single-crystalline elastic properties of different phases with (ii) microstructure-specific homogenization methods in order to predict macroscopic experimentally-detectable elastic parameters of new materials (see Figure 1). This scale-bridging scheme that directly links the atomistic and macroscopic levels allows for (i) systematic scanning of numerous chemical compositions via high-throughput quantum-mechanical calculations; and thus (ii) pre-selection of the most promising materials candidates. Consequently, a significant reduction of experimental costs and time is achieved when designing new materials with application-determined properties. The TGMD strategy has been recently successfully applied, e.g., for the development of ultra-light Mg-Li binary alloys optimized with respect to multiple conflicting criteria, such as elastic stiffness, shear modulus, and bulk modulus [33,34,35] and existing multi-component materials with complex microstructures in order to shed more light on (i) the structure-property paradigm [36]; and (ii) the robustness of fundamental composite-designing principles [37].

In contrast to many top-down approaches that start from the macroscopic scale and continue downscale, a quantum-mechanics-based bottom-up approach is chosen within the TGMD, both to identify more rapidly the most suited compositions with regard to the thermodynamic stabilization of the desired phase and to scrutinize some of the basic structural and mechanical features of possible alloy candidates (Figure 1). First, the thermodynamic stability for a variety of phases is determined in order to identify the stable one(s) as well as their volumetric ratio in a multi-phase alloy if necessary. Together with the thermodynamic stability of phases, the mechanical stability is tested by computing single-crystalline elastic constants. Second, polycrystalline elastic moduli (the shear modulus *G*, Poisson ratio *ν* or Young’s modulus *Y*) and other engineering parameters measurable at macroscale are predicted employing suitable homogenization techniques. These use the ab-initio predicted linear-elasticity parameters in conjunction with Hooke’s law as constitutive material laws and that allow scale-bridging between atomistic and macroscopic levels. Starting from an initial composition and based on the residuum/deviation of the properties on the macroscale, a new atomic composition is suggested and studied. This cycle is repeated until the desired properties are obtained. Following this strategy, an alloy composition with desired properties is obtained. If the properties are not accessible by any chemical composition, new phases, compositions or properties have to be identified or targets have to be modified or adapted.

The so-called ab initio (or first-principles) methods are the key modeling method within the TGMD strategy to calculate and to predict the thermodynamic quantities and the elastic constants. They are based on the fundamental laws of quantum mechanics and allow an accurate and parameter-free determination of a wide range of material parameters. Due to the large computational effort to solve the quantum-mechanical problem for each electron, these methods are restricted to limited system sizes (commonly up to a few hundred atoms). Despite this limitation, both thermodynamic and elastic properties of individual crystalline phases can be predicted and used within the TGMD approach.

In this paper, we use a self-consistent homogenization approach allowing to determine polycrystalline elastic moduli of not only single-phase aggregates but also multi-phase composites, taking into account information on thermodynamic stability of all present phases. Following the TGMD strategy, our approach is applied to materials design of Ti-alloys with bone-matching elastic properties. We proceed in four steps. First, the thermodynamic stability of the phases co-existing in the studied system is predicted (including chemical compositions and volumetric content of the phases) in a way that approximatively reflects the actual processing route of the samples in which a high-temperature state is quenched to exist at ambient conditions. Second, the single-crystal elastic stiffness data are determined for all the present phases. Third, the integral elastic stiffness of a single-phase polycrystalline aggregate is calculated. Finally, the theoretical data on phase stability and elastic properties of different phases are combined in order to estimate the homogenized elastic response of the multi-phase alloys. Specifically in case of Ti-Nb alloys, it is possible to go from a single structural phase (bcc, *β*) to a two-phase (hcp *α* and bcc *β*) material by changing the Nb content. The results are compared to the experimental elastic data obtained by ultrasonic testing.

## 2. Methodology

### 2.1. Ab Initio Calculations

All the parameter-free calculations were performed using density functional theory (DFT) [38,39] in the generalized gradient approximation (GGA) [40] employing Projected Augmented Wave (PAW) potentials [41] as implemented in the Vienna Ab-initio Simulation Package (VASP) code [42,43]. The plane wave cutoff energy was 170 eV. An 8 × 8 × 8 and a 10 × 10 × 6 Monkhorst-Pack mesh were used to sample Brillouin zones of bcc-based and hcp-based supercells, respectively.

**Figure 2:**
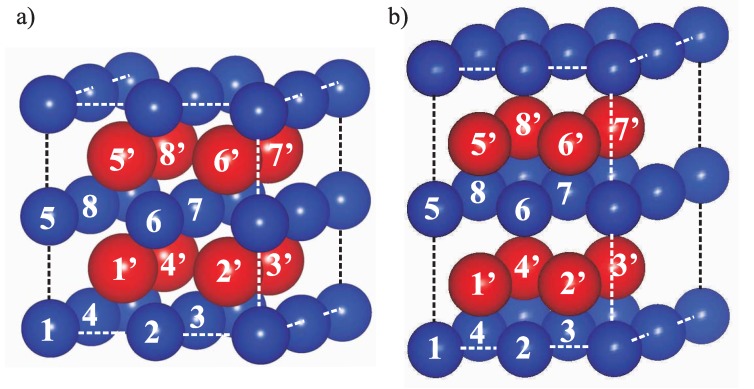
(**a**) The 2 × 2 × 2 16-atomic supercells used in the calculations of the cubic phase alloys; and (**b**) the hexagonal close-packed ones, with one half or the atoms (numbered 1–8) located in odd atomic layers and the second half (numbered 1’–8’) in even atomic layers (for sake of clarity depicted by larger red spheres) in the [001]${}_{\mathrm{bcc}}$ and [0001]${}_{\mathrm{hcp}}$ directions. The *β*-Ti-37.5at.% Nb alloys were modeled by two different ordered compounds with the Nb atoms located in positions marked in the figure by either numbers {124678} (below referred to as *β*-Ti-Nb${}^{I}$) or the set {1${}^{\prime }$24577${}^{\prime }$} (further referred to as *β*-Ti-Nb${}^{II}$).

The binary alloys were modeled using 2 × 2× 2 bcc (see Figure 2a) or hcp (Figure 2b) supercells. Each supercell contained a total of 16 atoms. A variety of ordered alloy compositions was sampled by replacing Ti atoms with Nb atoms. The lowest alloy composition was 6.25 at.% (one Nb or Ti atom in a 16-atomic supercell). For each alloy composition various local arrangements have been considered and studied spanning the whole concentration range (for further details see [44]). These calculations were used to identify the thermodynamically stable phases and the region of phase coexistence. For a sub-set of supercells exhibiting cubic symmetries, all three elastic constants (C${}_{11}$, C${}_{12}$ and C${}_{44}$) were calculated employing the methodology explained by, e.g., Chen *et al.* [45].

### 2.2. Analytic Homogenization Scheme

The integral elastic response of multi-phase polycrystalline samples can be determined from (i) the elastic single-crystalline constants; and (ii) the volumetric fractions of the components within a self-consistent *T*-matrix solution for the effective medium [46,47]. The *T*-matrix approach is based on the multiple scattering theory and was originally applied to determine elastic properties of single-phase polycrystals with cubic symmetry by Zeller and Dederichs [48]. This concept was generalized by Middya and Basu [46] to the case of single-phase crystals of non-cubic symmetry and further extended by Middaya *et al.* [47] to multi-phase composites.

Following the original approach by Zeller and Dederichs [48], the basic steps of the effective medium approach may be reviewed as follows. A macroscopically homogeneous effective medium that contains microscopic fluctuations may be characterized by an effective stiffness $C_{ijkl}^{*}$ defined by:

(1) $$\langle \sigma_{ij}\left(r\right)\rangle =C_{ijkl}^{*}\langle \epsilon_{kl}\left(r\right)\rangle$$

where $\sigma_{ij}\left(r\right)$ and $\epsilon_{kl}\left(r\right)$ are the local stress and strain fields at a point r, respectively; and the angular brackets denote ensemble averages. Assuming the aggregate is in equilibrium, the local stiffness field C(r) can be decomposed into an arbitrary constant part $C^{0}$ and a fluctuating part δC(r). The resulting local strain, *ϵ*, distribution can then be written (in a short-hand notation) as

(2) $$\epsilon =\epsilon^{0}+GT\epsilon^{0},$$

where $\epsilon^{0}$ and *G* are the strain and modified Green’s function of the medium defined by $C^{0}$; and the *T*-matrix is given by

(3) $$T=\delta C{\left(I-G\delta C\right)}^{-1},$$

where *I* is the unit tensor. Employing the local stress-strain relation and Equations 1 and 2, we get

(4) $$C^{*}=C^{0}+\frac{\langle T\rangle }{I+\langle GT\rangle }.$$

The exact evaluation of 〈T〉 and 〈GT〉 is impossible for realistic cases. However, by neglecting inter-granular correlations that may occur in some cases in the form of grain-to-grain position-orientation correlation functions, the *T*-matrix can be rearranged in terms of single-grain *t* matrices ($t_{\alpha }$) for each grain *α*

(5) $$T=\sum_{\alpha }t_{\alpha }=\tau .$$

Inserting Equation 5 into Equation 4 leads to

(6) $$C^{*}=C^{0}+\langle \tau \rangle {\left(I+\langle G\tau \rangle \right)}^{-1}.$$

For a single phase polycrystal, the self-consistent solution of Equation 6 can be obtained by choosing a $C^{0}$ that satisfies

(7) 〈τ〉=0.

For a multi-phase polycrystal, a solution to Equation 6 can be found by accounting for the volume faction and *τ* of each phase i ($v^{i}$ and $\tau^{i}$ respectively) [46] via

(8) $$\langle \sum_{i}v^{i}\tau^{i}\rangle =0.$$

The application of the method to both single-phase aggregates and multi-phase composites relevant to Ti-Nb alloys follows.

### 2.3. Single-Phase Aggregate

For a single-phase polycrystal with cubic symmetry, Equation 7 simplifies [46,47] to the following expressions for $B^{*}$ and $\mu^{*}$

(9) $$B^{*}=B_{0}$$

(10) $$8\mu^{*3}+\left(9B_{0}+4C^{\prime }\right)\mu^{*2}-3C_{44}\left(B_{0}+4C^{\prime }\right)\mu^{*}-6B_{0}C_{44}C^{\prime }=0.$$

in Equation 9, the three independent single crystal elastic constants $C_{11},C_{12},C_{44}$ define the single-crystal bulk modulus $B_{0}=1/3\left(C_{11}+2C_{12}\right)$, the tetragonal shear modulus $C^{\prime }=1/2\left(C_{11}-C_{12}\right)$ and trigonal shear modulus $C_{44}$.

For polycrystals with hexagonal symmetry, Equation 7 reduces to a set of coupled equations for $B^{*}$ and $\mu^{*}$

(11) $$\begin{array}{ccc} 0 & = & 3\left(K_{v}-B^{*}\right)-\kappa \Delta^{\prime \prime } \end{array}$$

(12) $$\begin{array}{ccc} 0 & = & \frac{M-6\mu^{*}-\nu \Delta^{\prime \prime }}{1-\kappa \Psi -9\gamma \left(K_{v}-B^{*}\right)+\left(1/3\right)\nu \kappa \Delta^{\prime \prime }}+\frac{12(C_{44}-\mu^{*})}{1-2\kappa (C_{44}-\mu^{*})}+\frac{12(C_{66}-\mu^{*})}{1-2\kappa (C_{66}-\mu^{*})} \end{array}$$

where

(13) $$\begin{array}{ccc} 9K_{v} & = & C_{33}+2\left(C_{11}+C_{12}\right)+4C_{13}, \end{array}$$

(14) $$\begin{array}{ccc} M & = & C_{11}+C_{12}+2C_{33}-4C_{13}, \end{array}$$

(15) $$\begin{array}{ccc} \Psi & = & C_{11}+C_{12}+C_{33}-3B^{*}-2\mu^{*}, \end{array}$$

(16) $$\begin{array}{ccc} \Delta^{\prime \prime } & = & C^{2}-B^{*}\left(M-6\mu^{*}\right)-6\mu^{*}K_{v}, \end{array}$$

(17) $$\begin{array}{ccc} C^{2} & = & C_{33}\left(C_{11}+C_{12}\right)-2C_{13}^{2}, \end{array}$$

(18) $$\begin{array}{ccc} C_{66} & = & \left(1/2\right)\left(C_{11}-C_{12}\right), \end{array}$$

(19) $$\begin{array}{ccc} \kappa & = & \frac{-3(B^{*}+2\mu^{*})}{5\mu^{*}\left(3B^{*}+4\mu^{*}\right)}, \end{array}$$

(20) $$\begin{array}{ccc} \nu /3 & = & -\frac{1}{3B^{*}+4\mu^{*}}, \end{array}$$

and $C_{11},C_{12},C_{13},C_{33},C_{44}$ are the single-crystal elastic constants of the hexagonal system.

### 2.4. Multi-Phase Composite

The elastic constants of a multi-phase polycrystal can be determined directly by coupling Equation 8 for $\tau_{44}^{i}$ and the $\left(\tau_{11}^{i}+2\tau_{12}^{i}\right)$ components of the *T*-matrix. For materials with cubic symmetry, these equations read

(21) $$\begin{array}{ccc} 5\tau_{44} & = & {\left(\frac{1}{C_{11}-C_{12}-2\tilde{\mu^{*}}}-\kappa \right)}^{-1}+3{\left(\frac{1}{C_{44}-\tilde{\mu^{*}}}-2\kappa \right)}^{-1} \end{array}$$

(22) $$\begin{array}{ccc} \tau_{11}+2\tau_{12} & = & \frac{3\left(C_{11}+2C_{12}\right)-9\tilde{B^{*}}}{3-(C_{11}+2C_{12}-3\tilde{B^{*}})}, \end{array}$$

where *κ* is defined in Equation 19 with $\tilde{\mu^{*}}$ and $\tilde{B^{*}}$ replacing $\mu^{*}$ and $B^{*}$. For materials with hexagonal symmetries, the equations read

(23) $$\begin{array}{ccc} 30\tau_{44} & = & \frac{M-6\tilde{\mu^{*}}-\nu \Delta^{\prime \prime }}{1-\kappa \Psi -9\gamma \left(K_{v}-\tilde{B^{*}}\right)+\left(1/3\right)\nu \kappa \Delta^{\prime \prime }}+\frac{12(C_{44}-\tilde{\mu^{*}})}{1-2\kappa (C_{44}-\tilde{\mu^{*}})}+\frac{12(C_{66}-\tilde{\mu^{*}})}{1-2\kappa (C_{66}-\tilde{\mu^{*}})} \end{array}$$

(24) $$\begin{array}{ccc} \tau_{11}+2\tau_{12} & = & \frac{3\left(K_{v}-\tilde{B^{*}}\right)-\kappa \Delta^{\prime \prime }}{1-\kappa \Psi -9\gamma \left(K_{v}-\tilde{B^{*}}\right)+\left(1/3\right)\nu \kappa \Delta^{\prime \prime }} \end{array}$$

where *κ* is defined in Equation 19; *ν* is defined in Equation 20; and $\Delta^{\prime \prime }$ in Equation 16. Here again, $\tilde{\mu^{*}}$ and $\tilde{B^{*}}$ replaces $\mu^{*}$ and $B^{*}$ in the equations for *κ*, *ν*, and $\Delta^{\prime \prime }$.

### 2.5. Homogenized Young’s Modulus and Poisson’s Ratio

Once $\tilde{\mu^{*}}$ and $\tilde{B^{*}}$ have been determined, the homogenized Young’s modulus ($\tilde{Y^{*}}$) and Poisson’s ratio ($\tilde{\nu^{*}}$) for (an elastically isotropic) polycrystal can be determined using standard elasticity relationships. The homogenized polycrystalline Young’s modulus is calculated via

(25) $$\begin{array}{ccc} \tilde{Y^{*}} & = & \frac{9\tilde{B^{*}}\tilde{\mu^{*}}}{3\tilde{B^{*}}+\tilde{\mu^{*}}} \end{array}$$

and the homogenized polycrystalline Poisson’s ratio is calculated using

(26) $$\begin{array}{ccc} \tilde{\nu^{*}} & = & \frac{3\tilde{B^{*}}-2\tilde{\mu^{*}}}{3(2\tilde{B^{*}}+\tilde{\mu^{*}})}\;. \end{array}$$

### 2.6. Experimental Methods

In order to compare the predictions with experimental data four Ti-Nb alloys (Ti-10at.%Nb, Ti-20at.%Nb, Ti-25at.%Nb, Ti-30at.%Nb) were melted, cast and homogenized at T = 1200 ${}^{\circ }$C. Characterization was done with optical and scanning electron microscopy (SEM) in conjunction with EDX (energy dispersive X-ray spectrometry) and EBSD (electron back scatter diffraction) as well as X-ray Bragg diffraction methods (see [44] for further details). The elastic properties were investigated by using an ultrasonic resonance frequency method (GrindoSonic).

## 3. Results and Discussion

Materials design of novel Ti-alloys for biomedical applications (such as implants) is essentially a multi-criteria optimization constrained by (i) the fact that all alloyed chemical elements should be biocompatible; and (ii) the final material should elastically match human bones as closely as possible. In order to fulfill the first condition, only a few so-called “vital” elements from the Periodic table ensuring biocompatibility can be used, e.g., Au, Ag, Ti, Zr, Nb, or Mo. The second criterion can be conveniently quantified in terms of the polycrystalline Young’s modulus. In order to reduce the stiffness of Ti-alloys, we use titanium (that under ambient conditions crystallizes in the hexagonal closed-packed (hcp) *α*-phase with high elastic stiffness, see Figure 2a) alloy-stabilized in the elastically softer cubic body-centered *β*-phase (Figure 2b). Such stabilization can be achieved by alloying with a *β*-stabilizing alloying element such as bcc refractory metals like Nb, Mo, V, or Cr. The reduced stiffness nevertheless comes at a price, specifically a lower thermodynamic stability of the *β*-phase at T = 300 K when reducing the concentration of the stabilizer. Therefore, a complex inter-connection between the thermodynamic stability and stiffness is expected and that makes the combined thermodynamic and elastic analysis within the TGMD scheme necessary.

**Figure 3:**
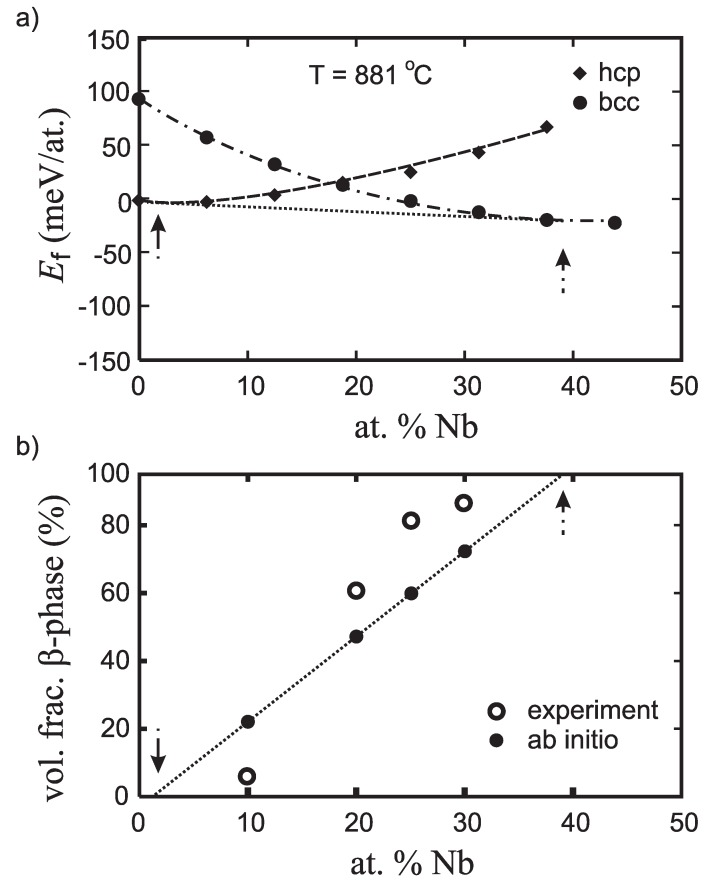
(**a**) Compositional dependence of the ab initio free energies of formation $E_{f}$ at T = 881 ${}^{\circ }$C for binary Ti-Nb alloys. The diamonds/circles mark $E_{f}$ for the hcp/bcc phase. The dotted line in part (a) shows the Gibbs construction that determined composition of both structural components of the composite (vertical arrows); (**b**) The compositional dependence of the thus predicted *β*-phase volume faction is shown in by full circles together with the experimental volumetric fractions (empty circles).

After checking six binary Ti-X alloys, niobium turned out to be the most promising element for stabilizing the *β*-phase (see also [44]). First, the thermodynamic stability of two-phase Ti-Nb alloys containing both bcc *β* and hcp *α* phases was studied. The details can be found in [44] while the main results are visualized in Figure 3. As a second step of the thermodynamic stability study, the composition of both present phases and their experimentally measurable volumetric fractions were determined. Using the compositional trends in the molar fraction from the Gibbs construction and density of phases (Figure 3a), the volumetric content of both phases has been theoretically predicted (see Figure 3b). The common tangent to energy curves of hcp and bcc phases defines the *α* phase to be essentially 100 at.% Ti and the *β* phase to be 37.50 at.% Nb and 62.50 at.% Ti. Figure 3b shows that the predicted volumetric content of the *β* phase is in good agreement with experimentally measured data [44]. The composite is predicted to consist of hcp *α*-phase with fairly low Nb concentration (under 4 at.%) and cubic *β*-phase with approximately 37.50 at.% Nb. As may be seen, the agreement between theory and experiment [44] is quite good.

After calculating thermodynamic properties of Ti-Nb alloys, the next step within the TGMD strategy consists in prediction of elastic properties. Computing the elastic properties of the Ti-hcp *α*-components is straightforward. However, the second phase, *β* Ti-Nb disordered solid solution, is more complex. In this case an exact distribution of Ti and Nb atoms over bcc-lattice positions is unknown. Therefore, the alloy *β*-Ti-37.5%Nb was modeled using two different ordered compounds (*β*-Ti-37.5%Nb${}^{I}$ and *β*-Ti-37.5%Nb${}^{II}$) that have the same composition but two different atomic arrangements exhibiting overall cubic symmetry of the 16-atomic supercells (see the Figure 2 caption). The theoretically predicted material properties used in this study are summarized in Table 1 and compared with the previous DFT calculations by Ikehata *et al.* [49,50] and experimental work by Reid [51].

It is important to note that some properties of *β*-Ti-37.5%Nb${}^{I}$ and *β*-Ti-37.5%Nb${}^{II}$ are very similar (for example the lattice constants difference is comparable to the computational error-bar) while other properties are not ($C_{44}$ differs by nearly factor of three). The difference in the formation energies of the two *β*-phase compounds is only about 12 meV/atom, indicating their nearly equal probability to occur in samples at elevated temperatures. Therefore, the elastic constants of the *β*-component of the α/β composite were modeled as a polycrystalline aggregate of two elastically anisotropic compounds, *β*-Ti-37.5%Nb${}^{I}$ and *β*-Ti-37.5%Nb${}^{II}$, with equal volume fraction of each constituent. The elastic constants of such elastically isotropic *β*-phase were computed by homogenizing the anisotropic elastic constants of *β*-Ti-37.5%Nb${}^{I}$ and *β*-Ti-37.5%Nb${}^{II}$ shown in Table 1 using Equation 8 and the set of coupled Equations 21 and 22. The resulting isotropic elastic constants of the *β*-phase were $B_{\beta }^{*}$ = 133 GPa and $\mu_{\beta }^{*}$ = 19 GPa.

**Table 1:** Theoretically predicted structural parameters and elastic constants of pure elements (Ti, Nb) and the cubic Ti-Nb compounds (*β*-Ti-37.5%Nb${}^{I}$, *β*-Ti-37.5%Nb${}^{II}$, for details see Figure 2) compared with available experimental data.

Material	*a*	c/a	$C_{11}$	$C_{12}$	$C_{13}$	$C_{33}$	$C_{44}$
Ti theory	2.921	1.585	200	72	90	191	40
Ti theory[49]	2.946	1.584	172	87	73	191	41
Ti experiment[52]	2.951	1.587	162	92	69	181	47
Nb theory	3.335	-	227	129	-	-	22
Nb theory[49]	3.325	-	247	134	-	-	15.6
Nb experiment[53]	3.301	-	246	133	-	-	28
*β*-Ti-37.5%Nb${}^{I}$ theory	3.261	-	156	121	-	-	10
*β*-Ti-37.5%Nb${}^{II}$ theory	3.264	-	168	118	-	-	29

Finally, employing Equations 8 and 21, 22, 23, 24), it is possible to estimate the elastic moduli of the polycrystalline α/β-composite. The five elastic components used for the hcp *α* phase were those calculated for pure hcp Ti (Ti theory in Table 1). These elastic constants were also homogenized using Equations 23,24 to determine $B_{\alpha }^{*}$ and $\mu_{\alpha }^{*}$. In this case, it was not necessary to assume that the homogenized *α* phase was elastically isotropic. Therefore, the anisotropic elastic constants for the *α* phase were $B_{\alpha }^{*}$ = 122 GPa and $\mu_{\alpha }^{*}$ = 50 GPa. The $B_{\alpha }^{*}$ value is in good agreement with our experimentally detected value of 115 GPa (at room temperature). The bulk modulus $B_{\alpha }^{*}$ is also in an excellent agreement with the value of 121 GPa obtained from numerical FEM simulations based on identical set of the ab initio calculated elastic constants (see the Appendix).

**Figure 4:**
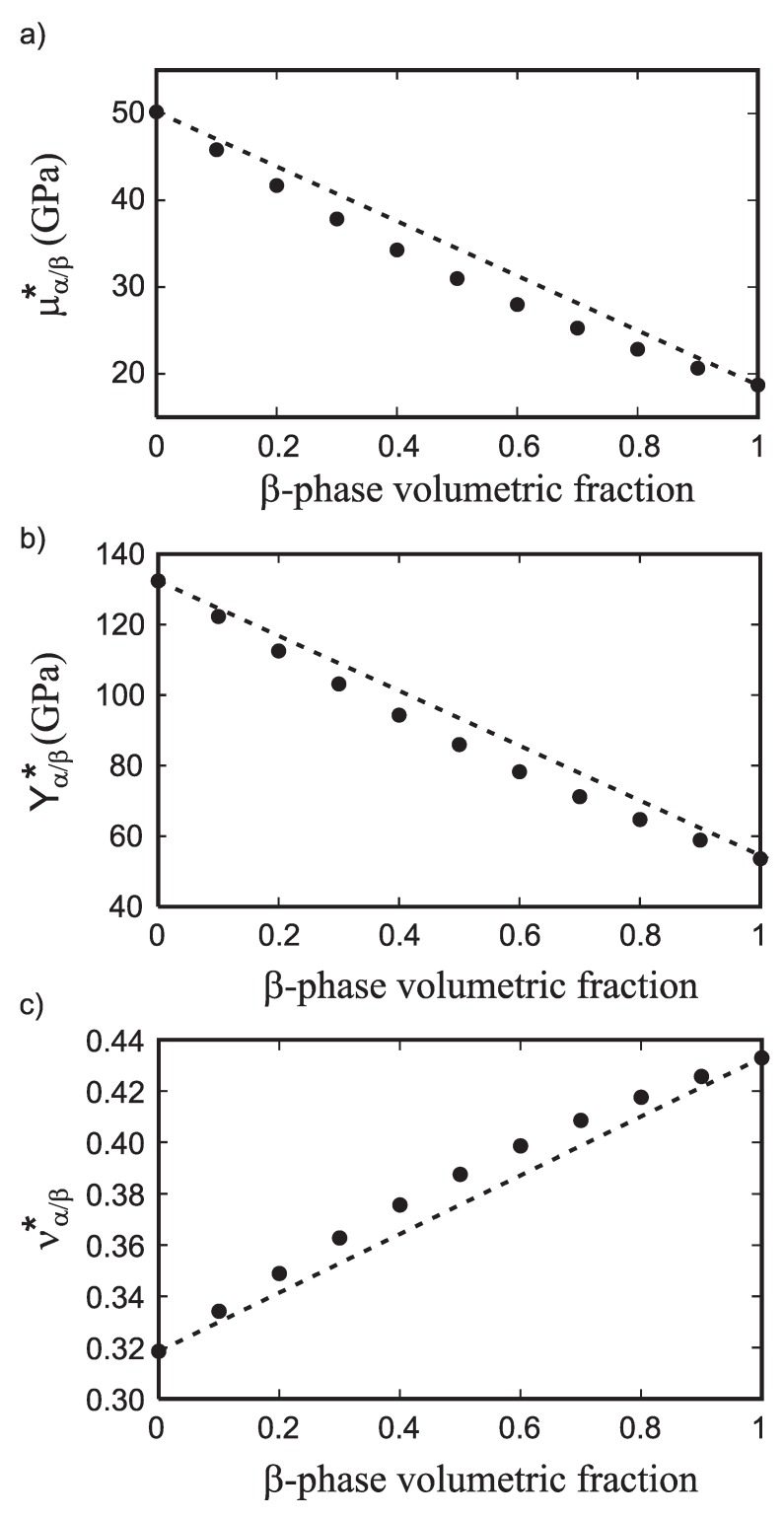
The theoretically predicted dependence of the (**a**) homogenized shear modulus $\mu_{\alpha /\beta }^{*}$; (**b**) Young’s modulus $Y_{\alpha /\beta }^{*}$; and (**c**) Poisson’s ratio $\nu^{{*}_{\alpha /\beta }}$ of the Ti-Nb composite as a function of the volumetric *β*-phase content numerically determined with 0.1 compositional step. The values are compared with the values obtained from a linear interpolation (dashed lines) between the *α* and *β* components.

The resulting dependence of the homogenized bulk modulus $B_{\alpha /\beta }^{*}$ as a function of the volumetric phase content has been found nearly identical to the linear interpolation between the $B_{\beta }^{*}$ = 133 GPa and the homogenized bulk modulus of pure hcp Ti polycrystalline component $B_{\alpha }^{*}$ = 122 GPa. In contrast, the homogenized shear modulus ($\mu_{\alpha /\beta }^{*}$), Young’s modulus ($Y_{\alpha /\beta }^{*}$), and Poisson’s ratio ($\nu_{\alpha /\beta }^{*}$) of the multiphase Ti-Nb alloys deviate from a simple linear trend as shown in Figure 4(a–c). Both $\mu_{\alpha /\beta }^{*}$ and $Y_{\alpha /\beta }^{*}$ display a negative deviation from linearity while $\nu_{\alpha /\beta }^{*}$ shows a positive deviation. These non-linear composition dependencies of the elastic constants illustrate the importance of using homogenization schemes. Figure 4a,b also shows that the overall shear and Young’s modulus increases as the amount of *α*-phase increases. This trend was expected since $\mu_{\alpha }^{*}>\mu_{\beta }^{*}$.

**Figure 5:**
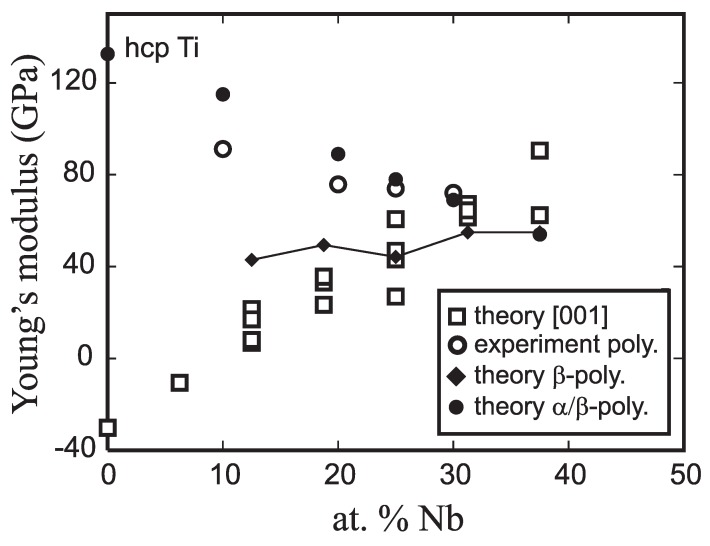
Predicted and experimentally obtained Young’s moduli. The theoretical single-crystal Young’s moduli for the soft [001] crystal direction of the cubic lattice cell are shown by empty squares. The homogenized Young moduli of hypothetical *β*-phase polycrystals with varying Nb content (not from the Gibb’s construction) are shown by full diamonds connected by a line. Full circles stand for the predicted $Y_{\alpha /\beta }^{*}$ and experimental data are visualized by empty circles.

In order to verify the theoretical predictions, a few Ti-Nb samples that had both *α* and *β* phases present were cast and there properties were probed [44]. The results are listed in Table 2 and a comparison of the experimental and theoretically predicted Young’s modulus is plotted in Figure 5. The results in Table 2 show that generally for a given alloy composition, the ab initio based thermodynamic analysis underestimates the volume fraction of *β* phase. Excluding the the Ti-10 at.% Nb sample, the errors in $v_{\beta }$ are in the order of 10%–20%. Underestimation of $v_{\beta }$ then leads to an overestimation of the homogenized Young’s modulus, which can also be seen in Figure 5. Better agreement with the experiment may be expected if vibrational entropy (as studied, e.g., in [54]) is included in the free energy calculations and used to calculate $v_{\beta }$, calculate the elastic constants, and estimate the DFT error bars [55].

**Table 2:** Theoretically predicted polycrystalline integral elastic parameters and phase-composition of Ti-Nb composites with selected Nb concentrations (of actually cast samples) together with the experimental data.

Material	$v_{\beta }^{\mathrm{theory}}$	$v_{\beta }^{\exp.}$	$B_{\alpha /\beta }^{*}$	$\mu_{\alpha /\beta }^{*}$	$Y_{\alpha /\beta }^{*}$	$Y_{\alpha /\beta }^{\exp}$
*α*-Ti	0	0	122	115	132	-
Ti-10at.% Nb	0.17	0.06	124	0.43	115	91
Ti-20at.% Nb	0.49	0.60	127	0.32	89	75
Ti-25at.% Nb	0.60	0.81	129	0.28	78	74
Ti-30at.% Nb	0.75	0.90	130	0.24	69	72
*β*-Ti-37.5at.%Nb	1	1	133	19	54	-

The importance of accounting for the dual-phase nature of Ti-Nb is illustrated in Figure 5 by plotting the compositional dependence of Young’s modulus for single crystal, polycrystalline, and dual-phase Ti-Nb. For *β*-phase, single crystal Ti-Nb, the dependence of the Young’s modulus in the softest [001]${}_{\mathrm{bcc}}$ direction has been plotted as a function of Nb content (open squares in Figure 5). The single crystal Young’s modulus is negative for alloys with low Nb-concentrations, indicating that these alloys suffering from the lack of *β*-stabilizer are mechanically unstable at low temperatures. As the Nb concentration increases, the single crystal Young’s modulus also increases, a trend that is completely opposite of that which is experimentally observed. Homogenizing the single crystal elastic constants into a 100% *β*-phase polycrystal does not correct this erroneous trend. The homogenized Young’s modulus calculated from different cubic ordered compounds with varying Nb content is shown for a few selected Nb concentrations in Figure 5 (filled diamonds). The overall trend is qualitatively better as the Young’s modulus increases only very slowly with increasing Nb-concentration. However, this trend still contradicts the experimental trends. Finally, the compositional dependence of the Young’s modulus for a dual-phase (hcp-*α* and bcc-*β*) polycrystal is shown in Figure 5 (open circles). While the effect of Nb content on the theoretical Young’s modulus is stronger than that observed experimentally, the predicted compositional trend is qualitatively correct: Young’s modulus decreases with increasing amounts of Nb.

The predicted compositional dependence of the elastic constants for dual-phase Ti-Nb polycrystals can be used as the first step in theoretically guided materials-design strategy to develop bone replacing materials that have a reduced elastic mismatch with respect to bone. From this analysis it can be seen that the Young’s modulus of hcp *α*-Ti can be reduced from around 132 GPa to around 70 GPa by adding 30 at.% Nb to form a two-phase α/β composite with the cubic *β*-phase. The theoretical predictions were verified by experimental measurements. As our results indicate that 70 GPa is the minimum that can be obtained by alloying on the atomistic level, a further reduction of the Young’s modulus seem to be possible only via (i) an processing optimization on the microstructural level in case of the studied binary systems; or (ii) higher order alloying that can suppress the presence of the stiff *α*-Ti phase. Employing our theory-guided materials design strategy, it will become possible to pre-select most promising Nb compositions by quantum-mechanical calculations and avoid casting and testing of large numbers of samples from the whole compositional range. This can be essential in both on-going and future development of Ti-Nb binary alloys [56,57,58,59], ternary materials [60,61,62,63,64,65,66,67] (including Ti-Al-V [68,69,70] and Ti-Nb-V [71,72]), as well as higher-order alloys [73,74,75,76,77,78,79,80,81,82] intended for bio-medical applications [83,84,85,86,87,88].

## 4. Summary and Conclusions

A multi-disciplinary approach was used to predict the polycrystalline elastic constants of a dual-phase Ti-Nb alloy. The approach combines a thermodynamic analysis with a self-consistent homogenization scheme that can describe phases with differing crystal structures. Thermodynamics provides the composition and volume fraction of the various phases, while homogenization estimates polycrystalline elastic constants from single crystal ones. All of the input values for this multi-scale approach originate from ab initio calculations, making this approach a strong tool in a theory-guiding materials-design strategy.

In this study, the thermodynamic analysis predicts the bcc-*β* phase composition would be 38 at.% Nb and 62 at.% Ti and that the hcp-*α* phase would be nearly 100 at.% Ti. Despite the fact that our theoretical thermodynamic analysis overestimates the volume fractions of *β* phase compared with those experimentally found, the predicted compositional trend is qualitatively correct. The resulting Young’s and shear modulus of polycrystalline α/β Ti-Nb decreased as the volume fraction of bcc-*β* phase (or Nb content) increases. Theoretically, a complete suppression of the presence of hcp *α*-Ti phase would result in a reduction of the Young’s modulus to about 54 GPa, which is predicted in case of a single-phase *β* Ti-Nb phase containing 38 at.% Nb. While the predicted modulus values are generally higher than that experimentally observed, the compositional trends are predicted correctly. From an alloy design perspective, we can conclude that in order to achieve maximum softness, the amount of the soft cubic *β*-phase should be optimized via keeping the amount of Nb high enough to ensure thermodynamic stability of the *β*-phase but low enough in order to avoid high stiffness that is typical for pure bcc Nb. The optimum amount of *β*-phase, which on one hand is thermodynamically stable but on the other hand does not contain too much Nb, is then limited to 30–40 at.% Nb, and the predicted Young’s modulus in this concentration range is around 70 GPa.

## 5. Appendix

As a test of performance of the used analytical mean-field homogenization method, we used Finite Element Method (FEM) calculations taking into account the anisotropic elasticity material routine within the commercial finite element code MSC.Marc200x. A polycrystal containing 96 square shaped grains (4 × 4 × 6) was meshed with quadratic brick elements. In order to tackle the fact that crystal containing 96 grains does not contain enough grains to simulate a “real” polycrystal, 5 different microstructures were generated and the average elastic properties are reported. The standard deviation of the 5 runs was relatively small indicating that the average values are reasonable. In order to assure solution convergence, each grain was meshed with 27 elements, resulting in a mesh with 2592 total elements. Each grain had a different, randomly determined orientation that defined the orientation of its elasticity tensor. Three random Euler angles were generated using a random number generator. These Euler angles were then transformed into a rotation matrix, which was used to rotate the elasticity matrix (*i.e.*, the elastic constants in each grain differed only by a rotation).

Elastic constants of the 96 grain polycrystal were determined by simulating a tensile test. A fixed displacement along the six grain directions in the polycrystal was prescribed while displacements on three other orthogonal planes was fixed to prevent the mesh from translating. The stress-strain response of the polycrystal was calculated from the reaction forces and displacements calculated by the FEM code. The elastic stress-strain response of five different polycrystals was simulated and the average Young’s modulus was then calculated. Using ab initio predicted elastic constants of hcp Ti, the FEM simulations predict the polycrystalline Young’s modulus to be 131 GPa. This value is in excellent agreement with 132 GPa obtained from the the used analytical mean-field homogenization method, thus validating its performance for texture-free aggregates.
